# Residual microcalcifications after neoadjuvant chemotherapy for locally advanced breast cancer: comparison of the accuracies of mammography and MRI in predicting pathological residual tumor

**DOI:** 10.1186/s12957-017-1263-8

**Published:** 2017-11-06

**Authors:** Yeong Yi An, Sung Hun Kim, Bong Joo Kang

**Affiliations:** 1 Department of Radiology, St. Vincent’s Hospital, College of Medicine, The Catholic University of Korea, 93, Jungbu-daero, Paldal-gu, Suwon-si, 16247 Gyeonggi-do Republic of Korea; 2 Department of Radiology, Seoul St. Mary’s Hospital, College of Medicine, The Catholic University of Korea, 222, Banpo-daero, Seocho-gu, Seoul, 06591 Republic of Korea

## Abstract

**Background:**

The aims of this study were to correlate residual mammographic microcalcifications after neoadjuvant chemotherapy (NAC) with pathological results and to compare the accuracy of mammography (MG) and magnetic resonance imaging (MRI) in predicting the size of residual tumors.

**Methods:**

The imaging findings and pathological results for 29 patients with residual microcalcifications after NAC were reviewed. We compared the agreement of the measured extent of residual microcalcifications based on MG and residual enhancement based on MRI with the residual tumor size based on pathology.

**Results:**

At final pathology, residual microcalcifications were malignant in 55.2% of cases and benign in 44.8% of cases. In 36% of non-pCR cases, the remaining microcalcifications were benign. Compared with the measurements of residual tumor obtained from pathology, MG showed poor agreement, and MRI showed moderate agreement, for the entire group (concordance correlation coefficient [CCC] = 0.196 vs. 0.566). Regarding the receptor status, the agreement of measurements obtained by MG was superior to that obtained by MRI (CCC = 0.5629, 0.5472 vs. 0.4496, 0.4279) for ER(+) and HER2(−) tumors. In ER(−) tumors, the measurements obtained by MG showed the lowest agreement with the pathological tumor size, which had the highest agreement with those obtained by MRI (CCC = − 0.0162 vs. 0.8584).

**Conclusions:**

Residual mammographic microcalcifications after NAC did not correlate with malignancy in 44.8% of cases. Residual microcalcifications on MG were poorly correlated with pathological tumor size, and MRI might be more reliable for predicting residual tumor size after NAC. Tumor receptor status affected the accuracy of both MG and MRI for predicting residual tumor size after NAC.

**Trial registration:**

CRIS, KCT0002281; registered 6 April 2015, retrospectively registered

## Background

Neoadjuvant chemotherapy (NAC) has been established as the standard treatment for inoperable or locally advanced breast cancer (LABC). Moreover, NAC has been increasingly used as one of the emerging treatment options for operable breast cancer. The advantages of NAC are that it facilitates breast-conserving surgery by reducing primary tumor burden and that it improves survival by treating micrometastasis [1–5]. The achievement of pathological complete response (pCR) after NAC is a known surrogate marker of disease-free survival as well as overall survival [6]. Therefore, an accurate assessment of response to NAC and residual tumor is important for planning the extent of surgery and predicting prognosis.

To date, breast magnetic resonance imaging (MRI) is the most accurate imaging method for assessing the extent of residual tumor after NAC than other imaging modalities [7–14]. However, MRI has limitations in evaluating the accurate extent of breast cancer associated with suspicious malignant microcalcifications on mammography (MG). Controversy remains as to whether all residual microcalcifications after NAC reflect residual tumor or whether changes in the number and pattern of microcalcifications represent a therapeutic response. Previous studies have shown that residual microcalcifications after NAC are not always correlated with residual tumor burden [15–20]. Residual microcalcifications can represent not only remnant malignant tumors but also necrotic tumor cell products in patients after treatment [21–25].

Accordingly, the aim of this study was to correlate residual mammographic microcalcifications after NAC with pathological results. We also compared the accuracy of MG and MRI in predicting the size of pathological residual tumors.

## Methods

### Study population

A prospectively, consecutively collected database was created based on 59 LABC patients with stage II or III who received NAC between April 2015 and April 2016 at our institution (*n* = 59). Institutional review board approval was obtained, and all patients provided written informed consent before inclusion in this study. All patients received four cycles of doxorubicin plus cyclophosphamide or six cycles of doxorubicin plus docetaxel chemotherapy. In HER2(+) patients, targeted therapy (Herceptin) was added to the chemotherapy. All patients were radiologically assessed by MG and MRI after chemotherapy and before surgery.

Based on a retrospective review of our prospectively maintained database, we identified 29 patients with suspicious malignant microcalcifications within the tumor bed on both pre- and post-NAC MG (*n* = 29). Twenty-seven patients not showing microcalcifications within the tumor bed were excluded (*n* = 27). Three patients were lost to follow-up during treatment and were also excluded (*n* = 3). The patients and tumor characteristics included in this study are noted in Table 1.

**Table 1:** Patient characteristics

	No. of patients (*n* = 29) (%)
Age	50.6 ± 9.0
Symptom	
No (abnormal screening)	21 (75.0)
Yes (palpable or discharge)	7 (25.0)
Clinical stage	
IIA	3 (10.3)
IIB	7 (24.1)
IIIA	18 (62.1)
IIIC	1 (3.5)
Chemotherapy regimen	
AC	9
AT	20
Operation method	
BCS	6 (20.7)
Mastectomy	23 (79.3)
Histologic type	
IDC	27 (93.1)
ILC	1 (3.5)
Mucinous carcinoma	1 (3.5)
Histologic grade	
Grade 1 or 2	18 (62.1)
Grade 3	11 (37.9)
ER status	
(+)	11 (37.9)
(−)	18 (62.1)
PR status	
(+)	14 (48.3)
(−)	15 (51.7)
HER2 status	
(+)	11 (37.9)
(−)	18 (62.1)
Molecular subtypes	
Luminal	18 (62.1)
HER2-enriched	10 (34.5)
Triple negative	1 (3.4)
Clinical response by MG	
PR	5 (17.2)
SD	23 (79.3)
PD	1 (3.4)
Clinical response by MRI	
CR	6 (20.7)
PR	18 (62.1)
SD	5 (17.2)
Pathological response	
pCR	4 (13.8)
Non-pCR	25 (86.2)

### Mammography and MRI examinations

Both MG and MRI examinations were performed in all 29 patients before and after NAC. Mammography was performed using Lorad Selenia (Hologic, Bedford, MA, USA) and Mammomat Inspiration (Siemens Medical Solutions Erlangen, Germany). Craniocaudal and mediolateral oblique mammograms were obtained for all patients.

The MRI examination was performed with the patient in the prone position using a 3.0-T scanner (Verio; Siemens Healthcare, Erlangen, Germany) equipped with a dedicated breast coil. The standardized protocol at our institution was performed in the axial plane and consisted of T2-weighted images, diffusion-weighted images, and dynamic series, post-processing subtraction, and maximal intensity projection images. For the dynamic contrast-enhanced studies, one pre-contrast and five post-contrast dynamic series were acquired using a T1-weighted flash three-dimensional (3D) VIBE sequence (TR/TE 4.4/1.7, flip angle 10°, 1.2-mm slice thickness without gap) before and at 10, 70, 130, 190, 250, and 310 s after an injection of 0.1 mmol/kg bodyweight of gadobutrol (Gadovist; Bayer Healthcare, Berlin, Germany).

### Radiological assessment by MG and MRI

MG and MRI findings were retrospectively reviewed by two radiologists with 6 and 14 years of experience in breast imaging, who were blinded to the histopathologic, clinical, and imaging findings of other modalities. Both the morphology and distribution of suspicious malignant microcalcifications within the tumor bed were classified according to the BI-RADS lexicon of MG [26]. To assess the change in the residual microcalcification extent, the maximal diameters of the microcalcifications were measured on pre- and post-NAC MG images. To assess residual lesions by MRI, the maximal diameter of the enhancing tumor on early post-contrast images of the post-NAC MRI was also measured and compared with that based on the pre-NAC MRI. When no residual enhancement was found, the size was set to 0 cm.

### Pathological assessment as a reference standard

To assess the pathological response to NAC, one pathologist with 17 years of experience in breast pathology evaluated the final surgical specimen obtained after a breast-conserving surgery or mastectomy. The tumor size, site, histological type, tumor grade, and expression of the estrogen receptor (ER), progesterone receptor (PR), and human epidermal growth factor receptor 2 (HER2); Ki-67 status according to immunohistochemistry; focality (number of foci and sizes of individual foci); presence of ductal carcinoma in situ (DCIS); resection margin involvement; and pTNM staging according to the 7th AJCC staging system as well as the presence of lymph node metastasis, lymphovascular invasion, and microcalcifications in neoplastic tissue or benign non-neoplastic tissue were included in the final pathological report. Pathological complete response (pCR) was defined as the absence of residual invasive cancer and DCIS^27^. The size of the residual tumor according to histopathology was defined as the sum of the maximal diameters of measurable invasive components as well as DCIS components.

### Statistical analysis

Lesion type; tumor grade; ER, PR, and HER-2 statuses; pathological responsiveness; the shape and distribution of microcalcifications; and changes of microcalcifications before and after NAC were compared between benign and malignant microcalcifications at final pathological examination of the surgical specimens using Fisher’s exact test, and significance was assumed for *p* values less than 0.05. The correlation between the extent of the residual microcalcifications as measured by MG, the size of the residual enhancement as measured by MRI, and the actual tumor size based on pathology were assessed based on the concordance correlation coefficient (CCC) regarding lesion type and receptor status. SAS version 9.4 (SAS institute, Cary, NC, USA) and MedCalc software version 13.1.2.0. were used for data analysis.

## Results

### Clinicopathological features and radiological findings

The clinicopathological features and radiological assessment of the treatment response in our study populations are described in Table 1. After NAC and surgery, four of 29 patients achieved a pCR (13.8%), and the remaining 25 patients had residual malignancy (86.2%). The clinical response assessed by pre- and post-NAC MG was a partial response (cPR) in 17.2% (*n* = 5) of patients, stable disease (cSD) in 79.3% (*n* = 23) of patients, and progression of disease in 3.4% (*n* = 1) of patients. No patients had clinical complete response (cCR) based on MG. The clinical response, as assessed by MRI, was cCR in 20.7% (*n* = 6) of patients, cPR in 62.1% (*n* = 18) of patients, and cSD in 17.2% (*n* = 5) of patients. Six patients with cCR on post-NAC MRI underwent breast-conserving surgery after mammography-guided wire localization for residual microcalcifications. Two patients (33.3%) had residual tumor, and the remaining four patients had pCR.

MG and MRI findings as well as the radiologically assessed residual tumor after NAC are described in Table 2. Mammographically, lesions presented as microcalcifications in only 24.1% (*n* = 7) of patients and microcalcifications with combined mass densities in 75.9% (*n* = 22) of patients. On pre-NAC MG, fine linear/linear branching shape (51.7%) and segmental distribution (48.3%) were the most common on pre-NAC MG. After NAC, most microcalcifications remained stable (*n* = 24, 82.8%); 10.3% of patients showed a decreased extent (*n* = 3), and 6.9% showed an increased extent (*n* = 2). The mean size of the remaining microcalcifications on pre-NAC MG was 6.0 ± 2.5 cm, which was 5.3 ± 2.3 cm on post-NAC MG. On MRI, lesions appeared as a mass in 55.2% (*n* = 16) of patients, nonmass enhancement in 31% (*n* = 9) of patients, and both in 13.8% (*n* = 4) of patients. The mean size of enhancement on pre-NAC MRI was 5.4 ± 1.9 cm and that of residual enhancement on post-NAC MRI was 2.6 ± 2.1 cm.

**Table 2:** Mammography and MRI findings and radiological assessment of residual tumor after NAC

	No. of patients (%)
Mammographic findings	
Lesion type	
Microalcifications only	7 (24.1)
Microcalcifications with mass	22 (75.9)
Shape of microcalcifications	
Amorphous	4 (13.8)
Fine linear/linear branching	15 (51.7)
Fine pleomorphic	10 (34.5)
Distribution of microcalcifications	
Grouped	8 (27.6)
Regional	7 (24.1)
Segmental	14 (48.3)
Pre-NAC microcalcifications size, MG (cm)	6.0 ± 2.5
Post-NAC microcalcification size, MG (cm)	5.3 ± 2.3
MRI findings	
Lesion type	
Mass	16 (55.2)
Nonmass	9 (31.0)
Both	4 (13.8)
Pre-NAC tumor size, MRI (cm)	5.4 ± 1.9
Post-NAC tumor size, MRI (cm)	2.6 ± 2.1

### Histopathological correlation of residual microcalcifications after NAC

Residual microcalcifications after NAC were correlated with the final pathological examination on the surgical specimen (Table 3). Residual microcalcifications after NAC were associated with residual malignancy in 55.2% (*n* = 16) of cases and with benign pathology in 44.8% (*n* = 13) of cases. Of 16 residual malignancies noted in the pathology report, six tumors had microcalcifications associated with invasive carcinoma and ten had microcalcifications associated with DCIS. Of 13 benign pathological lesions, five were microcalcifications associated with fibrocystic change, four with stromal fibrosis, one with atypical ductal hyperplasia, one with unusual ductal hyperplasia, one with a complex sclerosing lesion, and one with columnar cell change. In four patients with pCR, all residual microcalcifications showed benign pathologies. In 25 patients with non-pCR (86.2%), the residual microcalcifications were associated with malignancies in 16 (64%) and benign pathologies in 9 (36%). As described in Table 3, fine pleomorphic microcalcifications were significantly correlated with residual malignancy after NAC, and amorphous microcalcifications were correlated with benign microcalcifications after NAC (*p* = 0.015). Other mammographic features, including lesion type, distribution of microcalcifications on pre-NAC MG, and the change of microcalcifications on post-NAC MG, did not differ between malignant and benign calcifications.

**Table 3:** Residual mammographic microcalcifications after NAC correlated with final pathological results

	Benign calcifications (*n* = 13)	Malignant calcifications (*n* = 16)	*p* value
Lesion type			1.000
Microcalcifications only	3 (42.9)	4 (57.1)	
Mass + calcifications	10 (45.4)	12 (54.6)	
Shape of microcalcifications			0.015
Amorphous	3 (75.0)	1 (25.0)	
Fine linear/linear branching	9 (60.0)	6 (40.0)	
Fine pleomorphic	1 (10.0)	9 (90.0)	
Distribution			1.000
Segmental/regional	9 (42.9)	12 (57.1)	
Grouped	4 (50.0)	4 (50.0)	
Change of calcifications			0.486
Decrease	0 (0.0)	2 (100.0)	
Increase	2 (66.7)	1 (33.3)	
No change	11 (45.8)	13 (54.2)	
Pathologic responses			0.03
pCR	4 (100.0)	0 (0.0)	
Non-pCR	9 (36.0)	16 (64.0)	

### Agreement between the residual tumor size measured by MG and MRI and pathological tumor size

The mean sizes of the residual tumors measured on MG, MRI, and the pathological specimens at surgery were 5.3 ± 2.3 cm, 2.6 ± 2.1 cm, and 3.7 ± 2.6 cm, respectively. The agreement regarding the size of the residual tumor based on MG, MRI, and pathology is shown in Table 4. The CCC of the residual tumor size assessed by MG was poor and that by MRI was moderate (0.196 and 0.566, respectively) in all patients. With respect to receptor status, the agreement between the extent of residual microcalcifications and the size of the pathological residual tumor was highest in ER(+) and HER2(−) tumors, and this agreement was slightly higher than that for MRI. In ER(−) tumors, MG measurements showed the lowest agreement with the size of the pathological residual tumor, and MRI measurements showed the highest agreement (CCC = − 0.0162 vs. 0.8584).

**Table 4:** Residual tumor size and CCC between radiological measurements and pathology by immunoistochemistry

	Pathologic residual tumor size (cm)	MG residual microcalcifications (cm)		MRI residual enhancement	
		Size (cm)	CCC (95% CI)	Size (cm)	CCC (95% CI)
All patients	3.7 ± 2.6	5.3 ± 2.3	0.1962 (− 0.1087–0.4673)	2.6 ± 2.1	0.5660 (0.2997–0.7505)
ER status					
(+)	4.2 ± 2.6	4.7 ± 2.2	0.5629 (0.1622–0.8043)	2.5 ± 2.1	0.4496 (0.0982–0.7013)
(−)	2.9 ± 2.4	6.4 ± 2.1	− 0.0162 (− 0.2852–0.2552)	2.7 ± 2.1	0.8584 (0.5692–0.9586)
PR status					
(+)	4.4 ± 2.8	5.5 ± 1.9	0.1881 (− 0.2547–0.5657)	2.6 ± 2.3	0.4588 (0.0635–0.7296)
(−)	2.9 ± 2.0	5.1 ± 2.7	0.1683 (− 0.2173–0.5015)	2.5 ± 1.9	0.7920 (0.4810–0.9260)
HER2 status					
(+)	3.1 ± 2.1	5.2 ± 2.7	0.0323 (− 0.2986–0.3562)	2.2 ± 1.9	0.6606 (0.3303–0.8468)
(−)	4.7 ± 2.9	5.5 ± 1.6	0.5472 (0.1459–0.7939)	3.1 ± 2.4	0.4279 (− 0.0806–0.7597)

## Discussion

In our study, microcalcifications within the tumor bed after NAC were unchanged in 82.8% of patients; residual microcalcifications were malignant in 55.2% of patients and benign in 44.8% of patients, and these findings were similar those obtained in previous studies [15–18]. Additionally, we found that fine pleomorphic microcalcifications were significantly correlated with residual malignancy, but amorphous microcalcifications were correlated with benign microcalcifications according to the final pathology. However, there was no significant difference between malignant and benign microcalcifications regarding distribution or changes after NAC.

We found that the accuracies of both MG and MRI in predicting the size of the pathological residual tumor were insufficient in cases of residual microcalcifications after NAC. In the study, the size of the residual tumor with remaining microcalcifications after NAC tended to be overestimated by MG and underestimated by MRI. The size of residual enhancement on post-NAC MRI was correlated to a greater degree to the size of the pathological residual tumor than to the extent of residual microcalcifications on post-NAC MG (CCC = 0.196 vs. 0.566). These findings are similar to those of Weiss et al. [18], who reported that the CCCs of MG and MRI with pathology were − 0.12 and 0.55, respectively.

We also found that the accuracies of both MG and MRI in predicting pathological tumor size were affected by tumor receptor status (Table 4). The agreement between the residual microcalcifications and the size of the pathological residual tumors was highest for ER(+) and HER2(−) tumors (CCC = 0.5629 and 0.5472, respectively) and lowest for ER(−) tumors (CCC = − 0.0162). The reliability of MRI in predicting the size of the pathological residual tumor was highest (CCC = 0.8584) for ER(−) tumors and substantial in PR(−) and HER2(+) tumors (CCC = 0.7920 and 0.6606, respectively). These results indicate that patients with ER(−) tumors, even if extensive microcalcifications remain on post-NAC MG, can be considered candidates for breast-conservation surgery if there is no residual enhancement on post-NAC MRI. In contrast, in patients with ER(+) or HER2(−) tumors, the residual microcalcifications should be completely resected because MRI might underestimate the residual lesions. Recently, Kim et al. [19] demonstrated that the reliabilities of both MG and MRI for the prediction of residual tumor differed among breast cancer subtypes. In all subtypes, the agreement between the extent of residual microcalcifications and the size of the pathological residual tumor was fair and was lower than that obtained using MRI (ICC = 0.368 and 0.723, *p* < 0.0001). The reliability of MG for the prediction of residual tumor was highest for the HR+/HER2+ subtype (ICC = 0.417) and lowest for the triple negative (TN) subtype (ICC = 0.205). The extent of residual tumor as measured by MG was more frequently underestimated in HR+/HER2(−) and TN tumors and was overestimated in HR+/HER2+ and HR−/HER2+ tumors. Although the statistical analysis methods that they used and their results differed from ours, both studies showed that the accuracies of both MG and MRI in predicting the pathological tumor size can be influenced by molecular subtype and receptor status. This means that the concern expressed about the incomplete resection of microcalcifications may depend on the subtype or receptor status of the breast cancer. Further investigations with a large, prospective cohort are needed to validate these results.

The presence of residual microcalcifications on post-NAC may confuse surgeons when they decide whether to perform breast-conserving surgery or mastectomy. Until now, there has been no consensus or guideline on how to handle residual microcalcifications after NAC. Feliciano et al. suggested that the complete resection of residual microcalcifications should remain the standard because the likelihood of residual malignancy remains high for patients who showed a decrease in microcalcifications on post-NAC MG and for those who showed a loss of enhancement on post-NAC MRI [17]. Neither the changes of microcalcifications on MG nor the combined assessment of MG and MRI after NAC predicted the presence of pCR. Although the absence of residual enhancement on MRI was strongly correlated with pCR, the discordant rate between absent residual enhancement on post-NAC MRI and pCR was 26%. In our study, the absence of residual enhancement on post-NAC MRI did not sufficiently predict the absence of residual tumor in 33% of cases. Therefore, we agree with the suggestion of Feliciano et al. that all microcalcifications within the tumor bed should be completely excised, although not all residual microcalcifications on post-NAC MG reflect residual malignancy.

The main limitation of our study was the small sample size. Additionally, the intra- and inter-observer variabilities between the measurement of residual lesions using MG and MRI were not evaluated. Third, our study population mostly included luminal cancers or HER2-enriched cancers. Therefore, the generalization of our results to all subtypes of breast cancer may be difficult. Fourth, patients who did not show microcalcifications within their tumor were excluded from this study. Because triple-negative cancer usually presents as a mass with a relatively well-circumscribed margin rather than as suspicious malignant microcalcifications, most triple-negative cancers were excluded from this study. Furthermore, these considerations explained the low pCR rate of 13.8% found in our study because previous studies have shown a higher pCR rate after NAC in TN cancer [27, 28].

## Conclusions

Most microcalcifications within the tumor bed were unchanged after the NAC, and residual microcalcifications did not correlate with residual malignancy in 44.8% of cases. Among mammographic features, the shape of microcalcifications within the tumor bed significantly differed between benign and malignant microcalcifications according to final pathology. The amorphous microcalcifications more frequently became benign, while fine pleomorphic microcalcifications were more frequently correlated with residual malignant microcalcifications. The extent of residual microcalcifications as measured by MG had poor agreement with the size of the pathological residual tumor, which was lower than that by MRI. The accuracies of both MG and MRI in predicting residual tumor size after NAC depended on tumor receptor status, which might impact surgical planning or concerns about the incomplete excision of residual microcalcifications in subsets of patients with breast cancer. However, further investigations with a large, prospective cohort are needed to validate these results. Although MRI provided more reliable information about residual tumor than MG, all residual microcalcifications should be completely excised because the absence of residual enhancement on post-NAC MRI did not sufficiently predict the absence of residual tumor in 33% of cases.

## Abbreviations

CCC: Concordance correlation coefficient
cCR: Clinical complete response
cPR: Clinical partial response
cSD: Clinical stable disease
ER: Estrogen receptor
HER 2: Human epidermal growth factor receptor 2
LABC: Locally advanced breast cancer
MG: Mammography
MRI: Magnetic resonance imaging
NAC: Neoadjuvant chemotherapy
pCR: Pathological complete response
PR: Progesterone receptor
TN: Triple negative
